# Pancoast Tumor as the Initial Presentation of a Metastatic Colon Adenocarcinoma

**DOI:** 10.7759/cureus.13371

**Published:** 2021-02-16

**Authors:** Ana Cunha, Miguel Quintela, Cláudia Costa, Armin A Quispe-Cornejo, Margarida Freitas-Silva

**Affiliations:** 1 Department of Internal Medicine, Centro Hospitalar Universitário São João, Porto, PRT; 2 Department of Clinical Hematology, Instituto Português de Oncologia do Porto, Porto, PRT; 3 Department of Endocrinology and Nutrition, Instituto Português de Oncologia do Porto, Porto, PRT; 4 Department of Intensive Care, Erasme University Hospital, Brussels, BEL

**Keywords:** pancoast tumor, colon adenocarcinoma

## Abstract

A Pancoast tumor is a rare condition, representing 3% to 5% of all lung cancers. The particular location of these lesions leads to the invasion of structures in the thoracic inlet, causing a constellation of symptoms known as Pancoast-Tobias syndrome. Diagnosis can be challenging due to their low prevalence and the possibility of being asymptomatic.

Most of these tumors are non-small cell lung cancers. However, rare conditions might arise at the same location, and histologic confirmation is relevant.

We report the case of a 45-year-old man admitted to the internal medicine department with a one-month history of night sweats. A full-body computed tomography (CT) scan revealed a mass on the upper lobe of the left lung, with soft tissue invasion. Histopathologic examination revealed an adenocarcinoma pattern originating from the colon. Colonoscopy showed two synchronous lesions. Hitherto, this is the second case ever described of a Pancoast tumor as metastasis of colon adenocarcinoma.

## Introduction

A Pancoast tumor, also known as a superior sulcus tumor (SST), is most commonly associated with non-small cell carcinoma of the lung (NSCLC) and poor outcomes [1]. It is diagnosed based on symptoms and radiologic features. Symptoms such as relentless, severe shoulder and arm pain related to the innervation of the eighth cervical and the first two thoracic nerve trunks are seen. Radiologic findings are mainly characterized by a mass or opacity in the apex of the lung, infiltrating the first two ribs (frequently T3 and T4) [2]. The etiological diagnosis in 90% of the cases is confirmed by percutaneous CT-guided fine-needle biopsy (FNB) [3]. Apart from NSCLC, other lesions may, although less frequently, result in Pancoast syndrome.

We describe a case of a patient who presented with a Pancoast tumor as metastasis of colon adenocarcinoma.

## Case presentation

**Figure 1:**
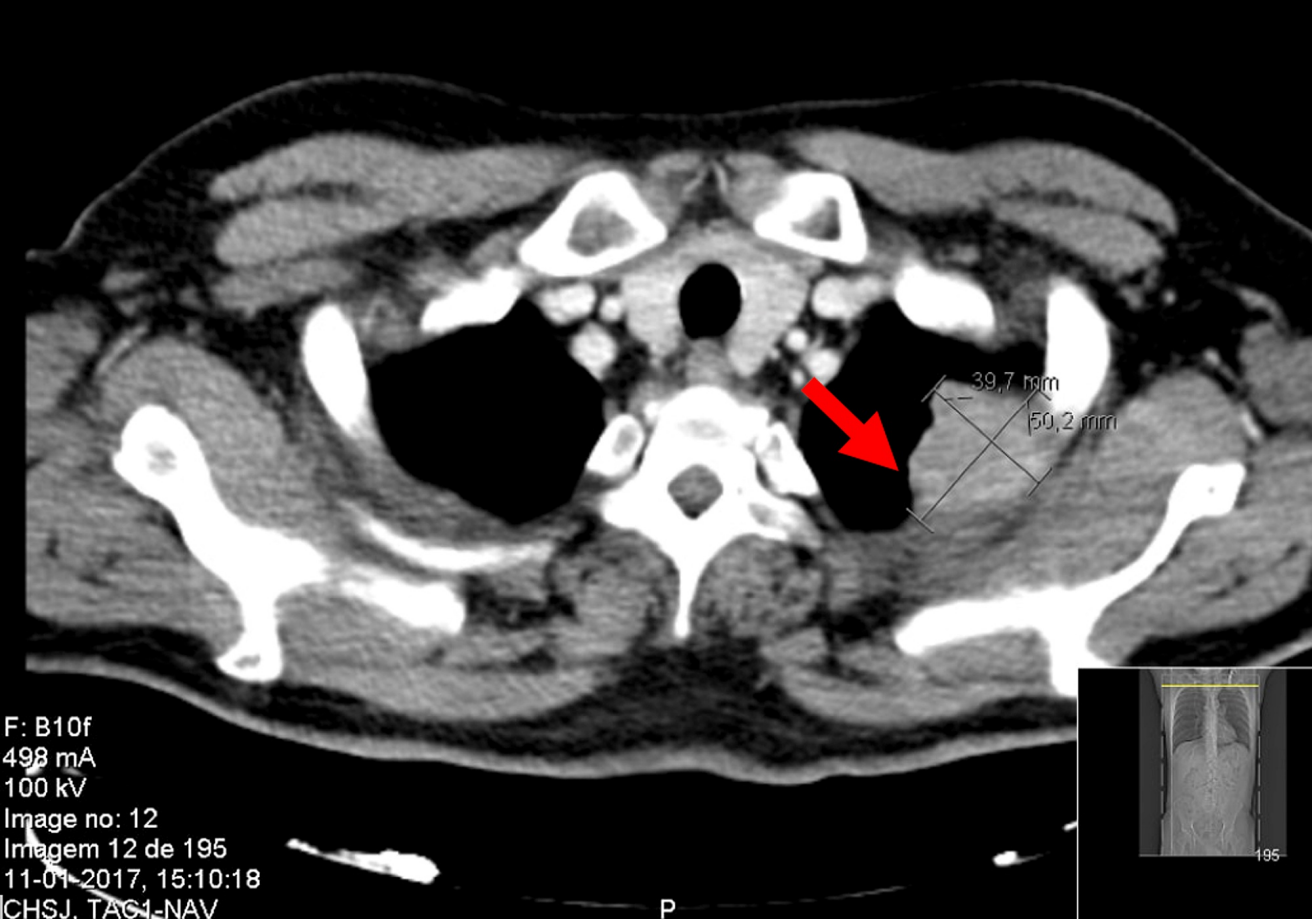
CT scan revealing an upper left lung mass of 40x50 mm, with the invasion of soft tissues within the second intercostal space

**Figure 2:**
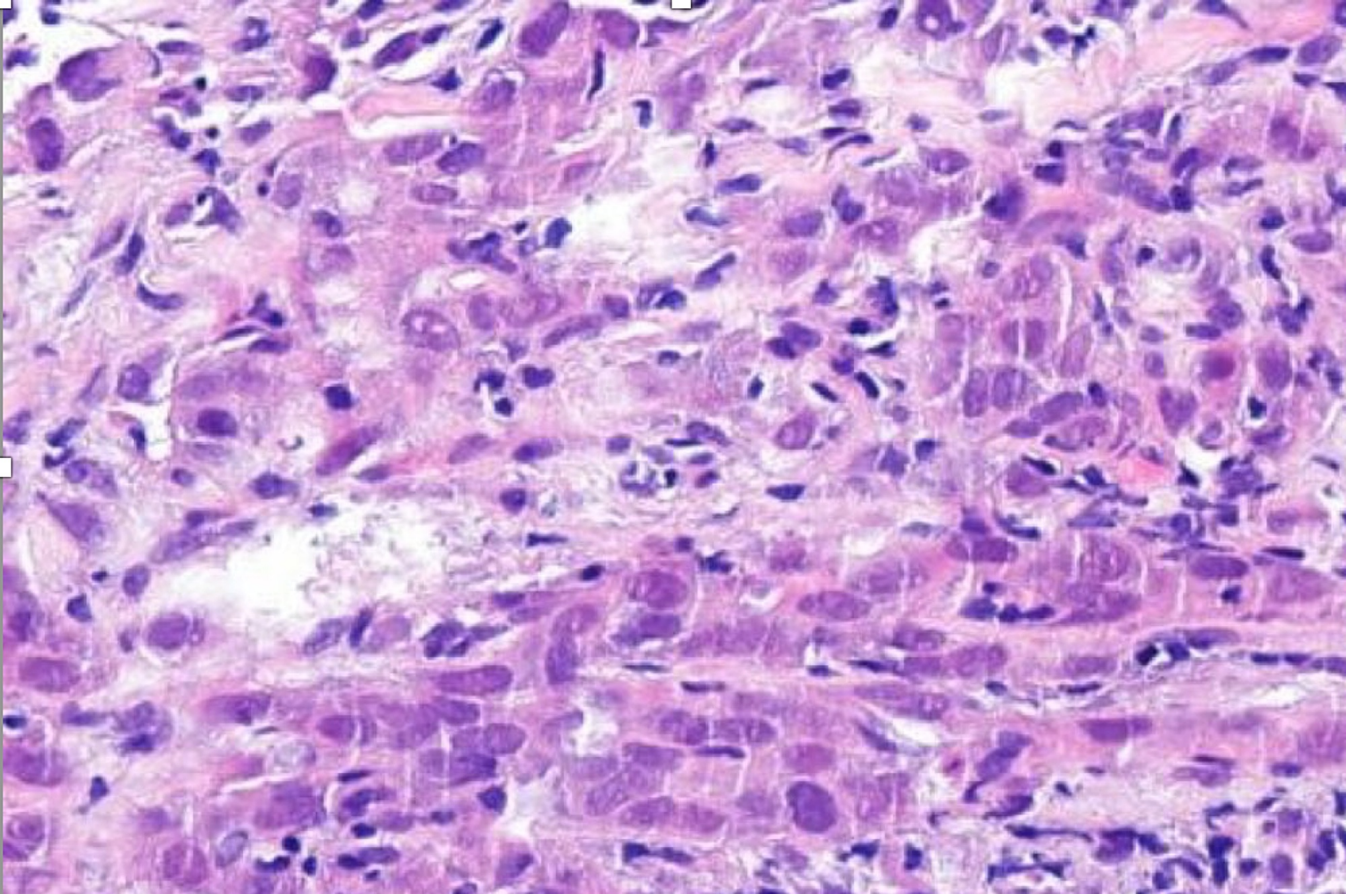
Pulmonary biopsy of malign neoplasm, which shows a trabecular pattern with the morphologic features of adenocarcinoma (400x)

A 45-year-old man was admitted to the hospital with a one-month history of night sweats. He had a history of heart failure due to alcoholic cardiomyopathy and smoking habits. There was no known relevant familiar history, as the patient was adopted. He denied anorexia and weight loss. He had no fever or other symptoms suggestive of infection. He denied pain in the upper body, namely, shoulder or arm pain. Physical examination was irrelevant apart from skin and mucous membranes paleness. Analytic evaluations reported hypochromic microcytic anemia with ferropenia (transferrin saturation <15%) and increased c-reactive protein (CRP; 43.5 mg/L (<3 mg/L)); white blood cell (WBC) count was among the referenced values. Human immunodeficiency virus (HIV), cytomegalovirus (CMV), Epstein-Barr virus (EBV) serologies, and Mycobacterium tuberculosis sputum culture were negative. Peripheral blood immunophenotyping reported normality. Anti-nuclear antibodies, antineutrophil cytoplasmic antibodies, and rheumatoid factor were negative, without complement consumption. A full-body CT scan revealed a 40 x 50 mm mass on the upper lobe of the left lung with the invasion of the apicoposterior bronchus of the upper lobe of the left lung and soft tissue invasion affecting the second intercostal space (Pancoast tumor) (Figure 1). Percutaneous transthoracic biopsy of the mass revealed a histological adenocarcinoma pattern (Figure 2).

**Figure 3:**
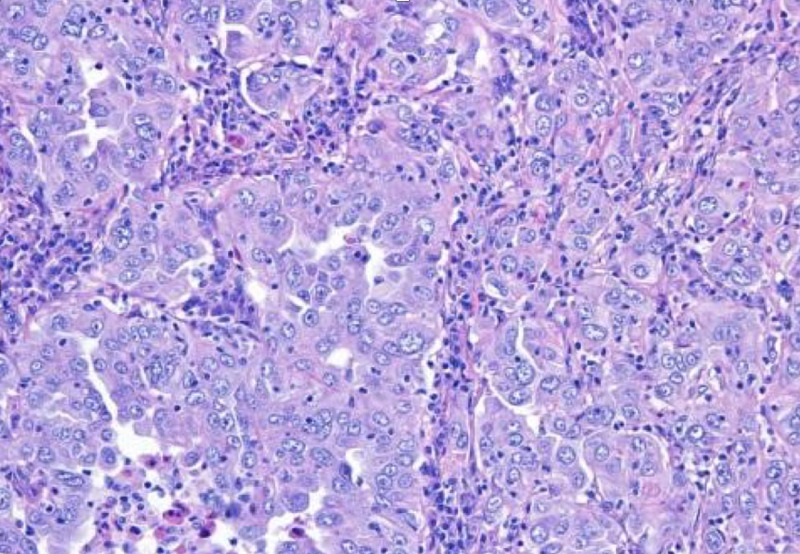
Colon biopsy with strong and diffuse expression of CK7 and weak and focal CDX2 in the absence of the expression of TTF-1 and CK20 (200x) CK7: cytokeratin 7; CDX2: caudal type homeobox 2; TTF-1: thyroid transcription factor 1; CK20: cytokeratin 20

Immunohistochemical (IHC) staining showed diffuse positivity for cytokeratin 7 (CK7), in the absence of thyroid transcription factor 1 (TTF-1), p63, cytokeratin 20 (CK20), and Napsin A, not allowing the clear identification of the origin of the tumor. A colonoscopy identified two small synchronous ulcerated lesions in the ascending and transverse colon. Histopathologic examination identified the same pattern found in the pulmonary mass (Figure 3), as well as focal positivity for caudal type homeobox 2 (CDX2). CDX2 was then sought and found in the pulmonary mass. The presumptive diagnosis of a stage IV ascendant and transverse colon synchronous adenocarcinoma with a solitary pulmonary metastasis (Pancoast tumor) was proposed.

**Figure 4:**
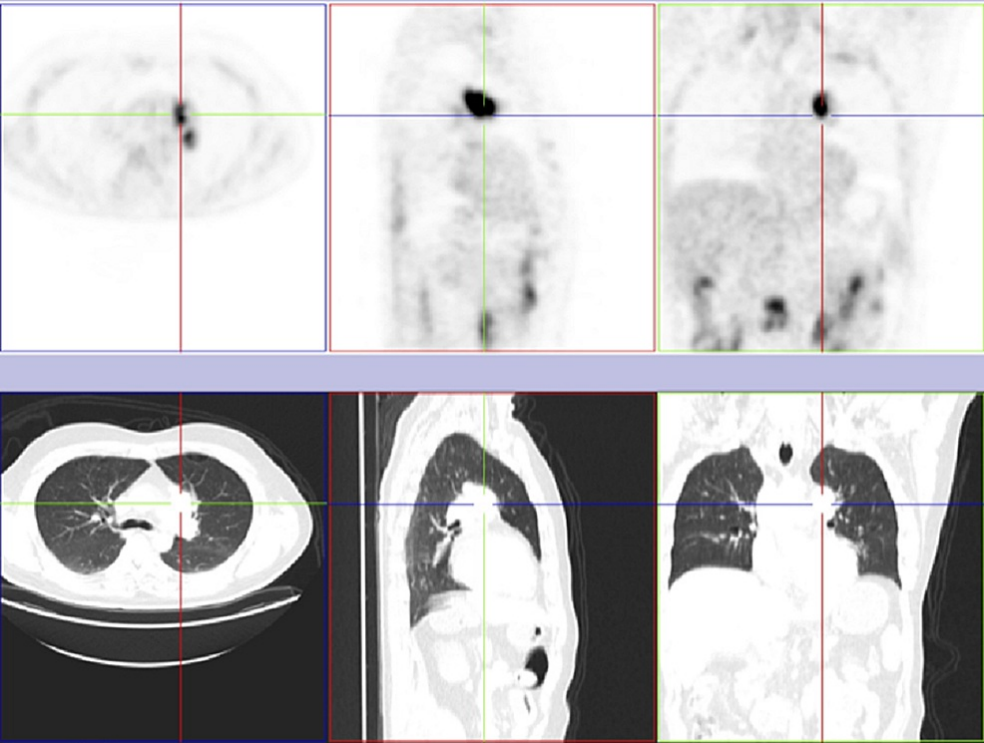
18F-FDG PET and CT scan showing the pulmonary lesion and aortopulmonary lymphadenopathies. 18F-FDG PET: 18F-fluorodeoxyglucose positron emission tomography

**Figure 5:**
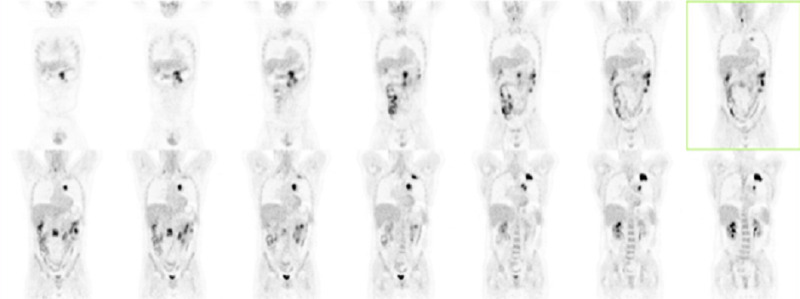
18F-FDG PET showing diffuse, multifocal intestinal uptake, with a higher signal in two foci: distal ascending portion and medium transverse 18F-FDG PET: 18F-fluorodeoxyglucose positron emission tomography

The 18F-fluorodeoxyglucose (18F-FDG) positron emission tomography (PET) scan showed a hypermetabolic focus in the left pulmonary apex, mediastinal lymph nodes, and multifocal intestinal uptake, with a higher signal in two foci: the distal ascending portion and medium transverse (Figure 4 and Figure 5).

An elective colectomy was proposed for anemia control due to the patient’s blood transfusion dependence. Besides colonic tumors, several implants along the jejunum were observed during the surgery. Therefore, a right hemicolectomy with concomitant excision of 100 cm of jejunum was performed. The pathology report revealed two neoplasms (one in the ascending colon and one in the transverse colon) on the right hemicolectomy specimen and 10 neoplasms on the segmental enterectomy specimen. The 12 neoplasms were identical and had the characteristics of poorly differentiated adenocarcinoma with moderate to abundant peritumoral and intratumoral lymphoid infiltrate, surface ulceration, and expansive growth. The two colonic neoplasms and eight enteric neoplasms had mucosal to subserosal invasion; the other two enteric neoplasms had muscular and serosa invasion, respectively. Images of the vascular, lymphatic, and venous invasion were identified. However, from the 32 lymph nodes retrieved, none had evidence of malignant cells.

The immunohistochemical study performed on multiple fragments of enterocolic neoplasms revealed weak and focal expression of CDX2 in the absence of expression of TTF1 and mucin 2 (MUC2). The cytogenetic study was negative for the epidermal growth factor receptor (EGFR) and anaplastic lymphoma kinase (ALK) genes, corroborating the diagnosis. Microsatellite instability was ruled out, and no additional genetic study was performed.

The tumor was classified as a Ki-ras2 Kirsten rat sarcoma viral oncogene homolog (KRAS)-wild type, and the patient was proposed to palliative chemotherapy with cetuximab and irinotecan.

He subsequently developed left thoracic and shoulder pain related to Pancoast tumor expansion, poorly controlled with opiates and paracetamol, for which symptomatic radiotherapy was initiated.

After six months of receiving immune chemotherapy, the progression of the disease was evident, with new-onset metastasis on the liver, peritoneum, and bone. Furthermore, the patient suffered from cancer-related (pulmonary thromboembolism) and treatment-related (mucositis) complications. The patient died 11 months after the initial diagnosis. 

## Discussion

Night sweats is an unspecific symptom that may represent a hypermetabolic state and is found in several pathologies such as infectious diseases or neoplasms [4]. We ruled out its most common related infections - HIV, viral hepatitis, tuberculosis, endocarditis - and autoimmune diseases. Endocrine disorders and substance abuse were also discarded. Since there was no organ-directed complaint, we performed a full-body CT scan. The Pancoast tumor was biopsied to define its primary or metastatic origin. Colonoscopy and colonic histopathology confirmed the primary neoplasm. A PET scan was also performed to exclude other metastases. Hemicolectomy, palliative chemotherapy, and radiotherapy were performed for symptoms control, and the patient died 11 months later.

Pancoast tumors are usually secondary to lung cancers (representing 3% to 5% of these cancers [1-2], especially NSCLC, squamous cell carcinomas [1], and adenocarcinomas [1,3]). Although rare, several other lesions develop in the superior sulcus, corroborating the necessity of a mass biopsy to establish diagnosis and guide treatment [3,5-6]. They include other tumors (primary chest wall and metastatic tumors, pleural mesothelioma, hematologic malignancies), infectious and inflammatory lung diseases (tuberculosis, aspergillosis, echinococcosis, bacterial lung abscesses, inflammatory pseudotumor, amyloid nodules), and vascular lesions (carotid pseudoaneurysm, hemangioma) that invade the apical bony skeleton of the thorax, producing a similar clinical and radiographic appearance [1]. Despite the initial high staging of Pancoast tumors (because of chest wall invasion), surgical resection and local radiotherapy can be performed either as a curative approach or as symptom management.

A Pancoast tumor metastasis from a distant neoplasm is uncommon, although metastatic lesions from the cervix, larynx, liver, bladder, and thyroid gland have been described [7-14]. A colorectal adenocarcinoma usually metastasizes to the lung by hematogenous pathways [15], usually creating a diffuse pattern. Therefore, the combination of Pancoast tumor metastasis of colorectal adenocarcinoma is exceptional. Up to now, this is the second case report describing this condition [16].

## Conclusions

This case illustrates a typical primary lung cancer mass that turned out to be of colonic origin in the context of smoking habits. Thus, it highlights exceptions and occurrences and reinforces an appropriate methodological and exhaustive medical practice to attain an accurate diagnosis.
